# Supplementary material for: Projected impacts of climate change on the range and phenology of three culturally-important shrub species
Source: PLoS One. 2020 May 8;15(5):e0232537. doi: 10.1371/journal.pone.0232537 (PMC7209123; doi:10.1371/journal.pone.0232537)
Supplement: S1 Table — (DOCX) [file pone.0232537.s001.docx]

**Table S1.** Mean thermal sums (sum of daily mean temperatures above 0 ℃), and earliest and latest observations of flowering and fruiting of beaked hazelnut, Oregon grape, and salal.

| Species | Flowering | Fruiting |  |
| --- | --- | --- | --- |
| Beaked hazelnut |  |  |  |
| Mean Thermal sum | 418 | 1914 |  |
| Earliest observation | 1/13/2015 | 3/14/2015 |  |
| Latest observation | 7/1/2014 | 12/13/2013 |  |
| Oregon grape |  |  |  |
| Mean Thermal sum | 460 | 1447 |  |
| Earliest observation | 1/17/2010 | 4/11/2004 |  |
| Latest observation | 7/6/1982 | 9/25/2008 |  |
| Salal |  |  |  |
| Mean Thermal sum | 1113 | 1790 |  |
| Earliest observation | 1/28/2012 | 5/25/2012 |  |
| Latest observation | 5/25/2012 | 11/20/2014 |  |
