# Supplementary material for: Projected impacts of climate change on the range and phenology of three culturally-important shrub species
Source: PLoS One. 2020 May 8;15(5):e0232537. doi: 10.1371/journal.pone.0232537 (PMC7209123; doi:10.1371/journal.pone.0232537)
Supplement: S1 Fig — These plots reflect the dependence of predicted suitability both on the selected variable and on dependencies induced by correlations between the selected variable and other variables. (DOCX) [file pone.0232537.s002.docx]

**
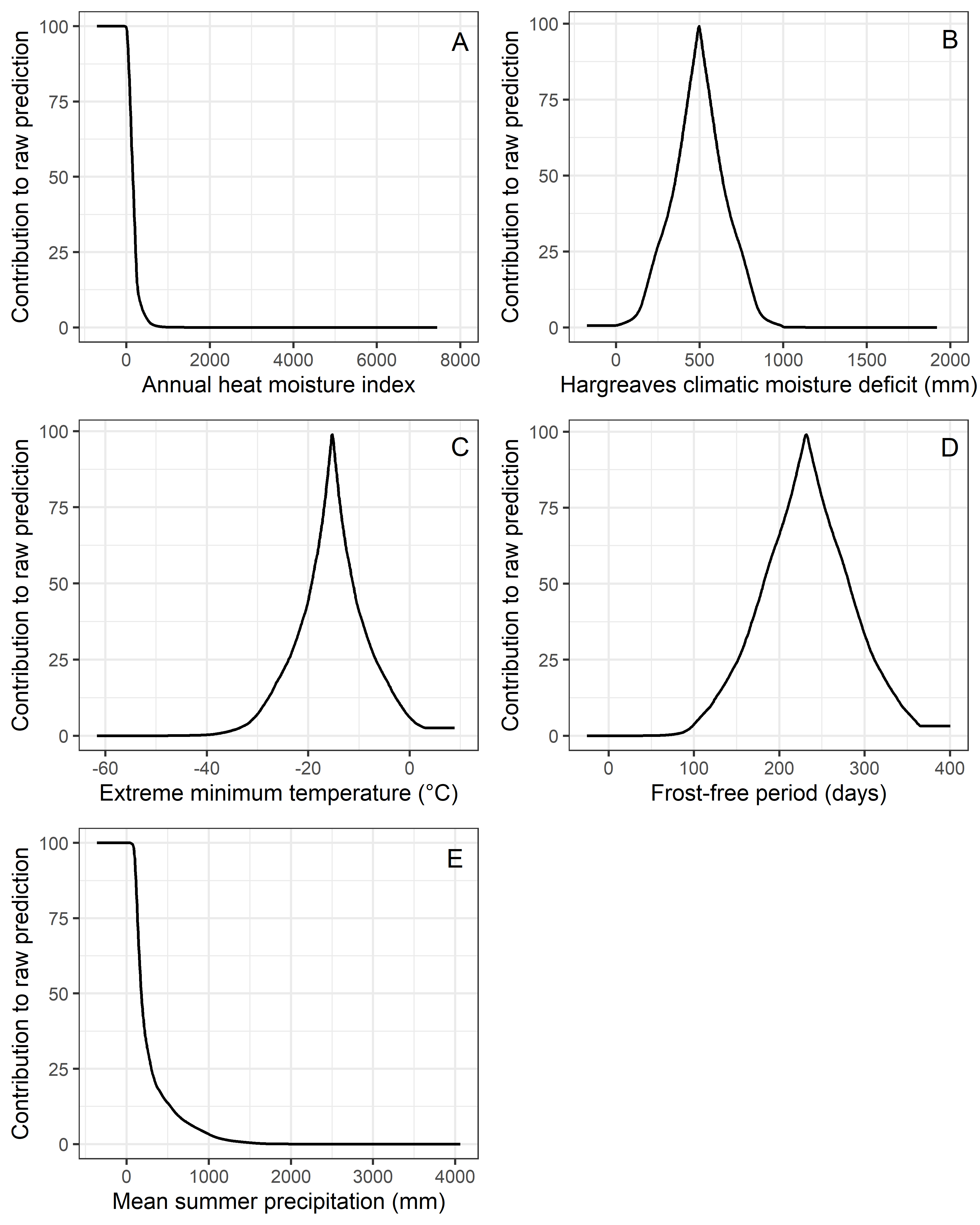
**

**Fig. S1.** Relationships between predicted habitat suitability and the 5 climatic variables in the best-fit species distribution model of hazelnut: (A) annual heat-moisture index, (B) Hargreaves climatic moisture deficit (C) extreme minimum temperature, (D) frost-free period, and (E) mean summer precipitation. These plots reflect the dependence of predicted suitability both on the selected variable and on dependencies induced by correlations between the selected variable and other variables.
