# Supplementary material for: Projected impacts of climate change on the range and phenology of three culturally-important shrub species
Source: PLoS One. 2020 May 8;15(5):e0232537. doi: 10.1371/journal.pone.0232537 (PMC7209123; doi:10.1371/journal.pone.0232537)
Supplement: S4 Fig — Background map used: World Terrain Base; data sources: Esri, USGS, NOAA; Republished under a CC BY license with permission from ESRI original copyright [2009]. (DOCX) [file pone.0232537.s005.docx]

**
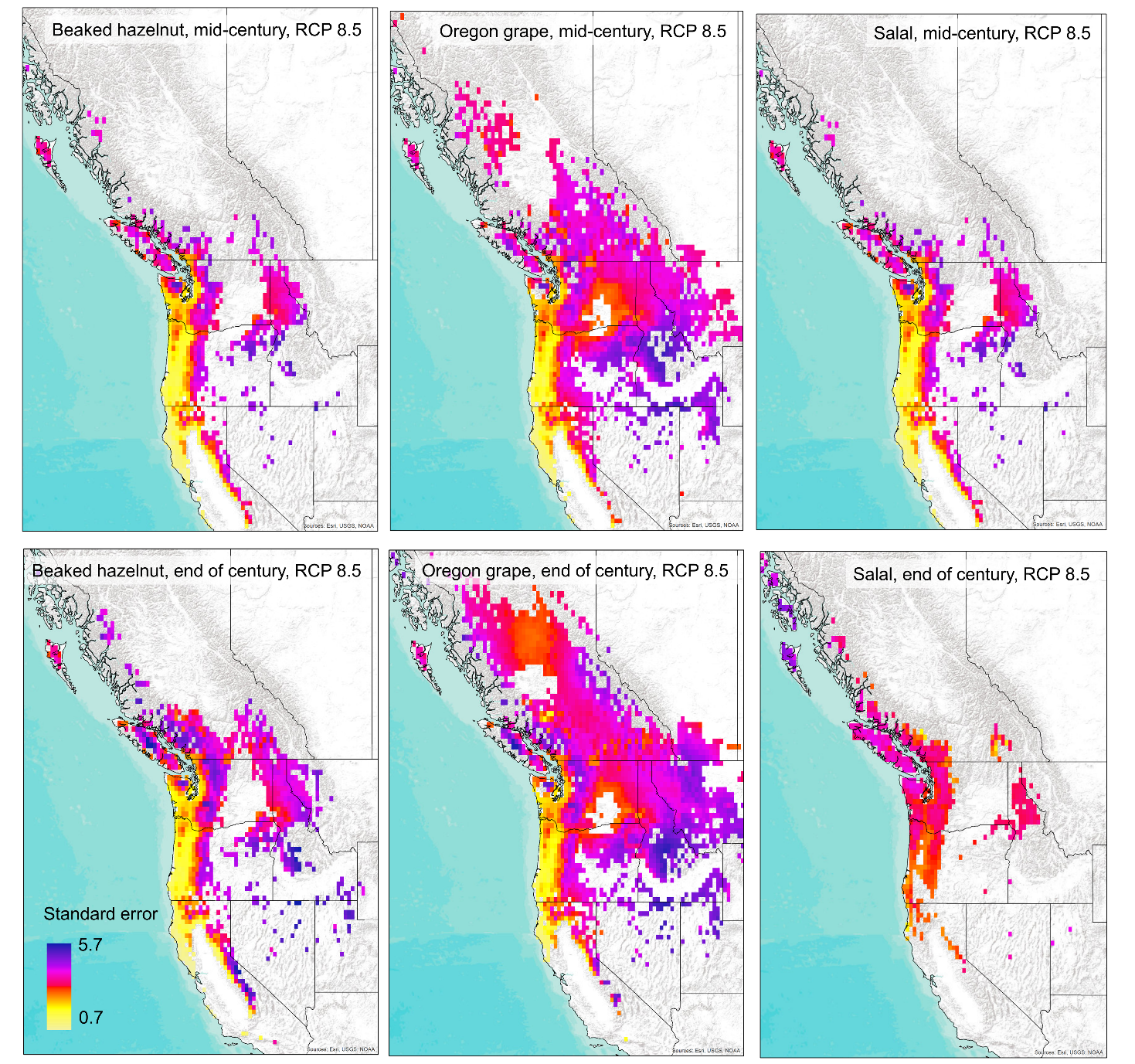
**

**Fig S4**. Standard error in projected flowering dates between 15 climate models across western North America under the RCP 8.5 emissions scenario for beaked hazelnut, Oregon grape, and salal by the mid-21^st^ century (top panels), and by the end of the 21^st^ century (bottom panels). Background map used: World Terrain Base; data sources: Esri, USGS, NOAA; Republished under a CC BY license with permission from ESRI original copyright [2009].
