# Supplementary material for: Projected impacts of climate change on the range and phenology of three culturally-important shrub species
Source: PLoS One. 2020 May 8;15(5):e0232537. doi: 10.1371/journal.pone.0232537 (PMC7209123; doi:10.1371/journal.pone.0232537)
Supplement: S1 Data — (PDF) [file pone.0232537.s007.pdf]

esri THE SCIENCE OF WHERE™

March 27, 2020

Requestor:

Janet Prevey

U.S. Geological Survey, Fort Collins Science Center

USGS, Reston, Virginia

E-mail: **janetprevey@gmail.com**

Publisher:
PLOS ONE

PLOS ONE:
Environmental Systems Research Institute, Inc. (Esri) grants to Frontiers in Microbiology a personal, nonexclusive, non-transferable, royalty-free, license and permission to use, copy, reproduce, and publicly display the Esri content (hereinafter the "Images") as set forth:

- Image used: Created using ArcGIS Online (Exhibit 1-6), World Terrain baselayer
- For use in upcoming PLOS ONE journal publication: "*Projected impacts of climate change on the range and phenology of three culturally-important shrub species*"
- For electronic distribution worldwide, published June 2020

Attribution line to be placed below Images or credit attribution section of article: "Content is the intellectual property of Esri and is used herein with permission. Copyright © 2020 Esri and its licensors. All rights reserved."

PLOS ONE is granted use of the Images as stated above. Esri warrants that it has the authority and right to grant permission of use of the Images, and that the Images and written description(s) do not infringe on any proprietary rights of third persons or contain any information that is unlawful, libelous, or in violation of any person's right to privacy and/or publicity. Esri reserves the right to grant permission for any other use of the Image.

Use of the Images is contingent upon proper copyright attribution being provided to Esri.

Regards,

Rhesha Moreau, IP Administrator
Contracts and Legal Department

380 New York Street
Redlands, California 92373-8100 USA

909 793 2853
info@esri.com

esri.com

<u>Exhibit 1</u>

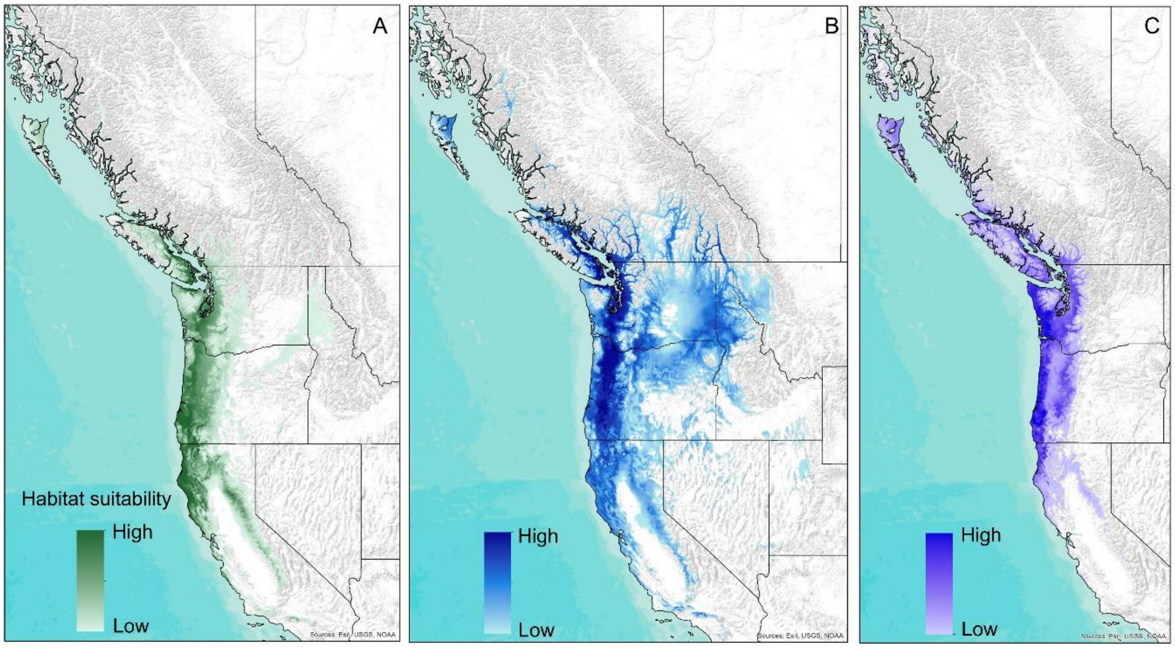

Fig. 1. Habitat suitability models for the recent time period (1981-2010) for (A) beaked hazelnut, (B) Oregon grape, and (C) salal. Background map used: World Terrain Base; data sources: Esri, USGS, NOAA; Republished under a CC BY license with permission from ESRI original copyright [2009].

380 New York Street
Redlands, California 92373-8100 USA

909 793 2853
info@esri.com

esri.com

Exhibit 2

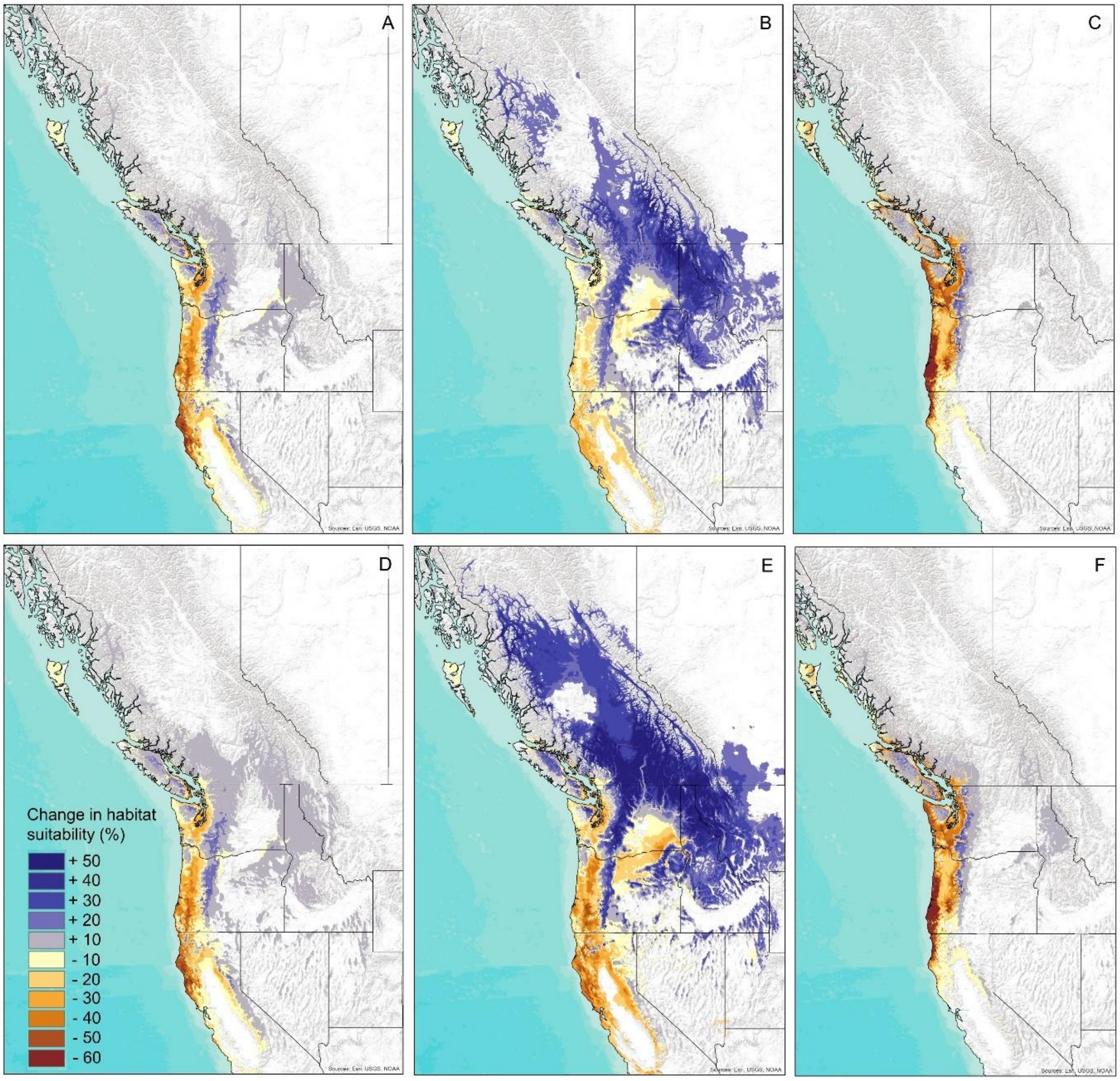

Fig. 2. Projected change in habitat suitability by the mid- 21st century under the RCP 8.5 emissions scenario for (A) beaked hazelnut, (B) Oregon grape, and (C) salal, and by the end of the 21st century under the RCP 8.5 emissions scenario for (D) beaked hazelnut, (E) Oregon grape, and (F) salal. Background map used: World Terrain Base; data sources: Esri, USGS, NOAA; Republished under a CC BY license with permission from ESRI original copyright [2009].

Exhibit 3

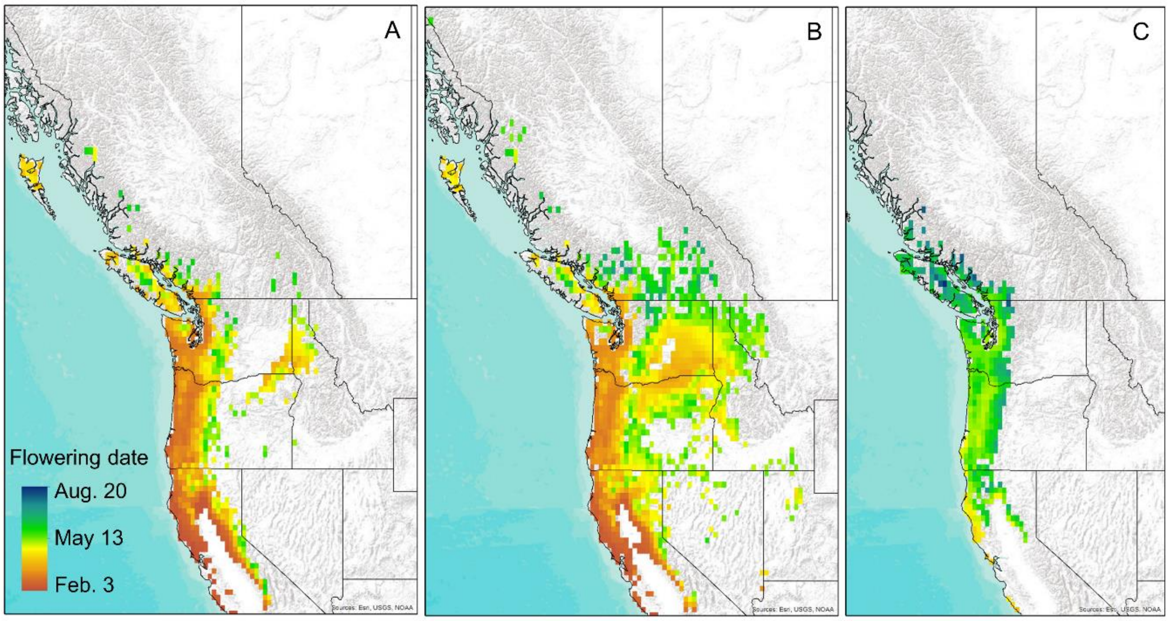

Fig. 3. Projected dates of flowering for the recent time period (1980-2010) for current ranges of (A) beaked hazelnut, (B) Oregon grape, and (C) salal. Background map used: World Terrain Base; data sources: Esri, USGS, NOAA; Republished under a CC BY license with permission from ESRI original copyright [2009].

Exhibit 4

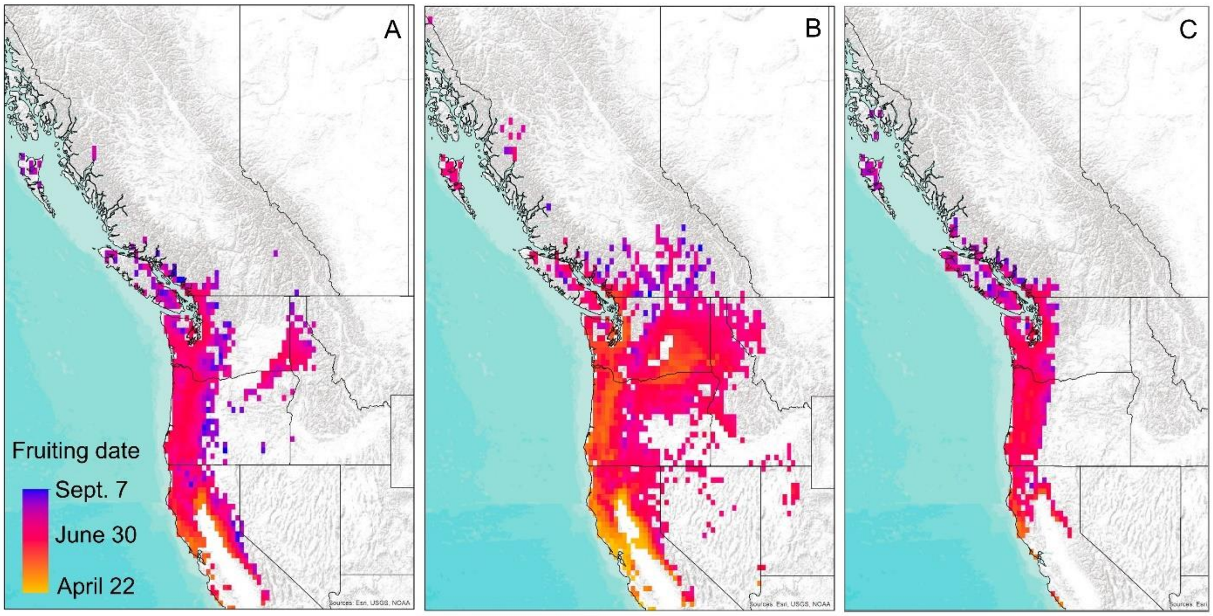

Fig. 4. Projected dates of fruiting for the recent time period (1980-2010) for the current ranges of (A) beaked hazelnut, (B) Oregon grape, and (C) salal. Background map used: World Terrain Base; data sources: Esri, USGS, NOAA; Republished under a CC BY license with permission from ESRI original copyright [2009].

Exhibit 5

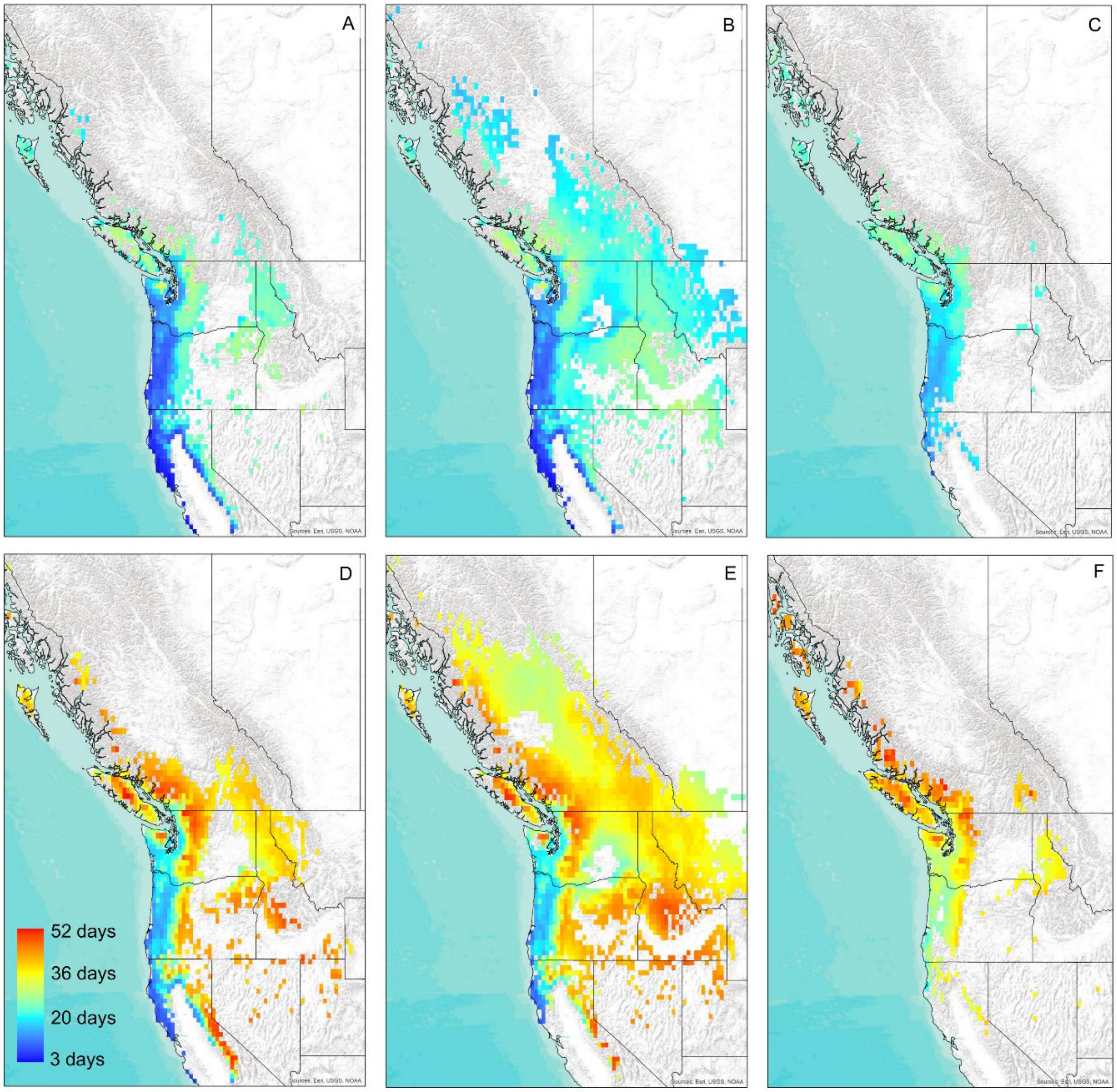

Fig. 5. Projected advance in flowering dates in future projected habitat by the mid- 21st century under the RCP 8.5 emissions scenario for (A) beaked hazelnut, (B) Oregon grape, and (C) salal, and by the end of the 21st century under the RCP 8.5 emissions scenario for (D) beaked hazelnut, (E) Oregon grape, and (F) salal. Background map used: World Terrain Base; data sources: Esri, USGS, NOAA; Republished under a CC BY license with permission from ESRI original copyright [2009].

380 New York Street
Redlands, California 92373-8100 USA
909 793 2853
info@esri.com
esri.com

Exhibit 6

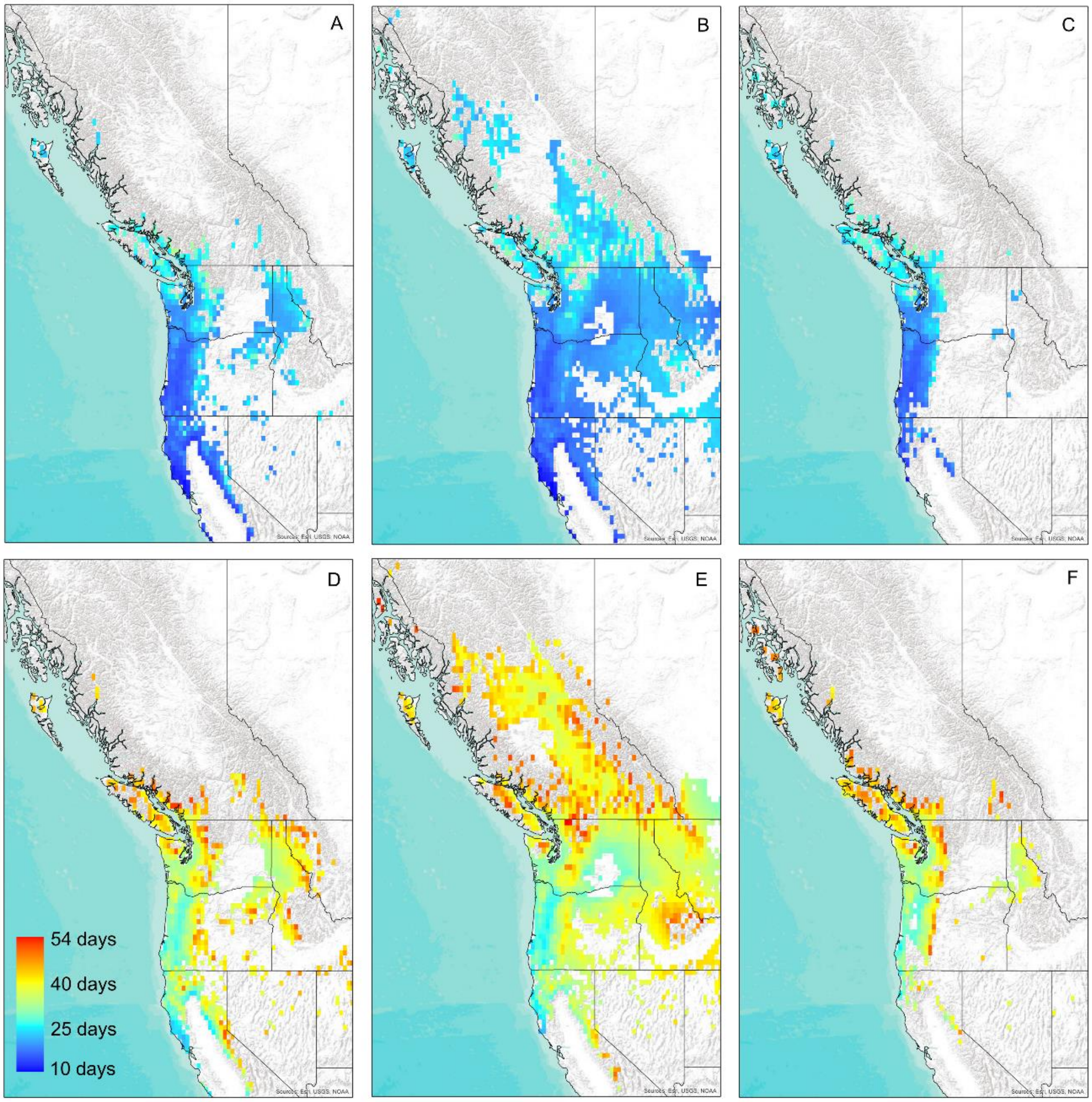

Fig. 6. Projected advance in fruiting dates in future projected habitat by the mid- 21st century under the RCP 8.5 emissions scenario for (A) beaked hazelnut, (B) Oregon grape, and (C) salal, and by the end of the 21st century under the RCP 8.5 emissions scenario for (D) beaked hazelnut, (E) Oregon grape, and (F) salal. Background map used: World Terrain Base; data sources: Esri, USGS, NOAA; Republished under a CC BY license with permission from ESRI original copyright [2009].
