# Supplementary material for: Projected impacts of climate change on the range and phenology of three culturally-important shrub species
Source: PLoS One. 2020 May 8;15(5):e0232537. doi: 10.1371/journal.pone.0232537 (PMC7209123; doi:10.1371/journal.pone.0232537)
Supplement: Attachment — Submitted filename: Three_shrubs_ResponsetoReviewers_final.docx [file pone.0232537.s008.docx]

**PONE-D-19-32397
Projected impacts of climate change on the range and phenology of three culturally-important shrub species
PLOS ONE**

**Dear Sergio Rossi and reviewers,**

**Thank you for your thoughtful edits and suggestions. We’ve addressed each point made by the editor and reviewers below, with our responses in *bold italic.* We’ve also highlighted all changes made to the manuscript in red in the ‘Revised Manuscript with Track Changes’ file.**

**Sincerely,**

**Janet Prevey, Lauren Parker, Connie Harrington**

**Editor and reviewer comments:**

Journal Requirements:

When submitting your revision, we need you to address these additional requirements.

1. Please ensure that your manuscript meets PLOS ONE's style requirements, including those for file naming. The PLOS ONE style templates can be found at <http://www.plosone.org/attachments/PLOSOne_formatting_sample_main_body.pdf> and <http://www.plosone.org/attachments/PLOSOne_formatting_sample_title_authors_affiliations.pdf>

***The manuscript meets the style requirements.***

2. We note that you have stated that you will provide repository information for your data at acceptance. Should your manuscript be accepted for publication, we will hold it until you provide the relevant accession numbers or DOIs necessary to access your data. If you wish to make changes to your Data Availability statement, please describe these changes in your cover letter and we will update your Data Availability statement to reflect the information you provide.

***We will add the DOI for the data as soon as the data is approved through the USGS data release review process, and prior to publication of the manuscript. No changes are needed for the data availability statement.***

3. Please upload a new copy of Figure 3 as the detail is not clear. Please follow the link for more information: [http://blogs.PLOS.org/everyone/2011/05/10/how-to-check-your-manuscript-image-quality-in-editorial-manager/](http://blogs.plos.org/everyone/2011/05/10/how-to-check-your-manuscript-image-quality-in-editorial-manager/)

***We’ve uploaded higher quality versions of all Figures, including Fig. 3.***

4. Please upload a copy of Figure 7, to which you refer in your text. If the figure is no longer to be included as part of the submission please remove all reference to it within the text.

***We’ve uploaded Fig. 7.***

5. We note that Figures 1-6 in your submission contain map images which may be copyrighted.

All PLOS content is published under the Creative Commons Attribution License (CC BY 4.0), which means that the manuscript, images, and Supporting Information files will be freely available online, and any third party is permitted to access, download, copy, distribute, and use these materials in any way, even commercially, with proper attribution. For these reasons, we cannot publish previously copyrighted maps or satellite images created using proprietary data, such as Google software (Google Maps, Street View, and Earth). For more information, see our copyright guidelines: <http://journals.plos.org/plosone/s/licenses-and-copyright>.

***All map images were created by the authors in ESRI’s ArcGIS 10.1; no copyrighted material was used. One can use static maps that include ArcGIS Online maps hosted by Esri in academic publications without the need for written permissions:*** [***https://doc.arcgis.com/en/arcgis-online/reference/static-maps.htm***](https://doc.arcgis.com/en/arcgis-online/reference/static-maps.htm)

***The one caveat is that the authors must provide attribution on or near the map or image that includes an ArcGIS Online map hosted by Esri. We have included this attribution in each panel of each Figure that uses an ESRI background map, and added this statement to the end of the Methods (l. 200-201): “All maps of climate envelope and phenology model results were created using ArcGIS® ArcMap 10.7.1 software (Esri, 2019).”***

5. Review Comments to the Author

Reviewer #1: It is important to understand habitat suitability, distribution, phenological mechanism and responses to climate change for the species with social and economic values, to inform assessment of future impact and management planning. This study applied MaxEnt and Thermal-sum models to estimate habitat suitability and flowering and fruiting phenology of three shrub species, which provided interesting results. More information should be stated to prove the reasoning of applied methods.

***We have added additional information to the Methods section explaining the reasoning for the applied methods we chose for species distribution modeling here: l. 105-106, and reasoning for why we chose thermal sum models here: l. 167-171).***

More evidence and discussion should be provided to prove the importance and how useful the research findings could be in this study.

***This research is directly applicable to climate impact statements being developed by many Tribes of the Northwest, as well as National Forests, National Parks, and other regional climate impact assessments. Our models provide an important new source of information on how climate might impact important understory species that have often been overlooked in previous climate assessments. We’ve added several specific examples for why these results are important and how these results can be used to the end of the discussion (l. 408-416).***

There are different phenology models for spring phenology including different sets of variables. This manuscript needs more arguments to state the reason why the authors selected thermal-sum model for flowering and fruiting phenology of the three shrub species.

***Thermal-sum models were used because our phenology observations were heterogeneous over time and space, and were not collected in a systematic matter at the same sites over time, thus it would be difficult to test a suite of models that include other, more complex, cues such as chilling and photoperiod. We were hesitant to test more complicated process-based models with these observational data as they may give misleading results (Hänninen et al., 2019). Additionally, thermal-sum, or growing degree day, models have been used extensively for horticultural and ecological studies, and have accurately predicted the flowering and fruiting dates of at least two shrub species (White et al., 2012; Laskin et al., 2019). While we are aware of the plethora of phenological models that incorporate multiple cues, such as chilling temperatures and photoperiod, we do not have any evidence that those cues are more important than forcing temperatures, and thus decided to use simple models with temperature sums. We’ve added this additional justification to the methods, (l. 167-171).***

***Citations:***

***Hänninen, H., Kramer, K., Tanino, K., Zhang, R., Wu, J., Fu, Y.H., 2019. Experiments Are Necessary in Process-Based Tree Phenology Modelling. Trends in Plant Science 24, 199–209. https://doi.org/10.1016/j.tplants.2018.11.006***

***Laskin, D.N., McDermid, G.J., Nielsen, S.E., Marshall, S.J., Roberts, D.R., Montaghi, A., 2019. Advances in phenology are conserved across scale in present and future climates. Nature Climate Change 9, 419–425. https://doi.org/10.1038/s41558-019-0454-4***

***White, S.N., Boyd, N.S., Van Acker, R.C., 2012. Growing Degree-day Models for Predicting Lowbush Blueberry (Vaccinium angustifolium Ait.) Ramet Emergence, Tip Dieback, and Flowering in Nova Scotia, Canada. horts 47, 1014–1021. https://doi.org/10.21273/HORTSCI.47.8.1014***

How much do we know about the phenological mechanism of the shrub species so far?

***There is not much known about the phenological mechanisms for shrubs in this region. However, for most temperate plant species, warming temperatures in spring are the most important cue for the timing of phenological events. The below graph shows the relationship between mean spring temperatures and our phenology observations for the three shrub species. As you can see, for all but one species*phenological event (hazelnut flowering) – there is a strong negative relationship between spring temperatures and the day of year that the phenological events occurred; that is, the warmer the spring temperatures, the earlier in the year flowering occurs. Again, we are not trying to assume that other environmental cues are not important for phenological timing, just that we currently do not have experimental evidence to justify including them in our models. Finally, we acknowledge that the physiological mechanisms driving the development of any of these shrub species are undoubtedly a complex interplay between multiple environmental drivers. Our methods and results are one effort to identify some of these drivers and quantify their relationships to shrub phenology.***

Are there any evidence supporting the thermal-sum model is the best for fitting flowering and fruiting phenology?

***Laskin et al. 2019 used thermal sum models to predict timing of fruiting of another fruit-producing shrub in the northwestern region, with landscape-scale thermal sum models performing very well and predicting the date of fruiting and leaf senescence of Sheperdia canadensis to within 3 days. Additionally, the above graph of our observations suggests that there is a relationship between warm temperatures and earlier phenological dates. Again, there is not much known about phenological mechanisms for shrub species, and we hope that this research will encourage more detailed studies using experimental data and repeat measurements to identify other possible mechanisms.***

If we know little of phenological mechanism, how can we use the future predictions based on the models with uncertain response mechanism?

***Our phenology models show how flowering and fruiting may advance with warmer temperatures, and we hope they can serve as a first step for future studies seeking to identify phenological mechanisms for shrubs, and monitor phenological changes over time. We have addressed the reasoning for the soundness of thermal-sum models in the Methods (l. 165-171), and have added additional statements about the limitations of our thermal sum models in the Discussion (l. 379-384). Ultimately all models will have some level of uncertainty, but we argue that given the close observed connection between temperature and phenology across wild and cultivated species, the future predictions give a reasonable assessment of likely changes and provide resource managers and other interested stakeholders with information useful for their planning purposes.***

The information of phenology data processing should be clear. How were all different phenology observation records collected in multiple sites incorporated into spatial grid cells?

***Daily temperatures for each phenology measurement were taken from the 1 x 1 km grid from Daymet for the year of that observation. Daily temperatures from these grid cells for each phenology observation were summed from January 1^st^ through the day of the phenological event in the year the observation was taken. We have clarified the methods to better explain the phenology data processing (l. 159-164).***

Were thermal-sum models fitted at grid cell level or site level or using the whole data?

***Thermal-sum models were calculated using all the data, as the sum of daily mean temperatures above 0 °C from January 1^st^ through the day each phenology observation from 1980-2017, and we used the mean value of all observations for flowering and fruiting to get thermal sum models for each species and phenological event. We have clarified the methods to better explain the phenology data processing (l. 159-164).***

In comparisons of current and future phenology, which values were used?

***In these comparisons, we compared the DOY of phenological events estimated for the year 2000 from Daymet data to phenological dates estimated for 2055 and 2085 using daily temperature data from each of 15 CMIP5 daily downscaled projections. We then calculated changes in phenological events based on the difference between current estimated DOYs and future projected DOYs of phenological events (l. 172-193).***

Yearly estimation was made, then calculated the mean date? E.g. mean phenological dates during 1981 and 2010 representing the current period? Or used one specific year estimation in comparisons?

***We used estimations from daily temperatures in the year 2000 to represent the current time period (l. 172-175).***

Line 374-379. Argument is weak. Relate to the comment above. Need to state the reason why this simple phenology model was used and how the limitations may affect the results and conclusions. While the model used growing-degree-day as the only driver of phenology and warming trend was the only driver of future change, the advanced results were straightforward. But is it realistic that flowering and fruiting time will be advanced without constrains over time? What important message should we take from the current results?

***We now expandd upon the reasoning that the simple phenological model was used in the methods (l. 167-172), and explicitly state how the results may be inaccurate if chilling temperatures or photoperiod are stronger environmental cues for phenology in the Discussion (l. 380-384).***

Line 23-24. Phenology means timing of biological events. Should be “the timing of critical biological events” or “phenology”

***Changed to “critical biological events”.***

Line 126. What are 16 bioclimatic variables? Need to refer to Table 2 here.

***We’ve added a reference to Table 2 (l. 125).***

Line 236-238. What do 2055 and 2085 time period mean? Are they yearly estimation in the two specific years or mean estimation for the whole time periods 2041-2070 and 2071-2100?

***2055 and 2085 indicated a 30-year period centered on these years. That is, the mean over 2041-2070 was indicated by 2055, and 2071-2100 by 2085. However, we have now changed the wording from 2055 and 2085 to mid- and end of the century respectively to clarify this, while indicating in the first use of these terms that “mid-century” is with respect to 2041-2070, and “end of century” is with respect to 2071-2100 (l. 118-119 and l. 237-239).***

Line 279-285. Could not find Figure 7.

***Apologies, we have now added Figure 7.***

Line 302. Need to explain how the results of least gain in habitat suitability suggested a decline of abundance of salal.

***We’ve altered the sentence to clarify: “Future climate projections show the least gain in suitable habitat for salal, in addition to declining habitat suitability through much of its current range.” L. 301-303.***

Line 308. Should specify “other factors”, such as population establishment and competition with other species.

***We have replace ‘other factors’ with population establishment and competition with other species (l. 312).***

Line 328-331. Should clarify the point from this sentence. Which results suggested more advanced fruiting of latest fruiting species than earliest fruiting species, and then shorten the time period between the earliest and latest fruiting.

***We have clarified this by referencing Fig. 6 and including the names of the species (Oregon grape and salal). “Our thermal-sum models show that with increasing accumulations of warm temperatures over the course of the summer, the later-ripening fruits of salal may ripen earlier (Fig. 6), and shorten the window of fruit availability between the earliest fruiting (Oregon grape) and latest fruiting species (salal), which could shorten the time-window of food availability for dispersers across the landscape.” l. 335-338.***

Line 352-354. Remind the specific periods of record for Oregon grape and salal here, so the readers would know how exactly short the observations record was.

***We now state the specific periods of record: “There were no significant trends in phenological timing for salal in the observations from Salem, Oregon, although the period of observations for salal was 18 years, much shorter than the 53 years of observations for Oregon grape.” L. 359-360.***

Line 372-373. Water availability and extreme weather events (e.g. drought and heat) may also affect phenology, which was rarely included in prior phenology models.

***We have added water availability and extreme weather events to the list of factors that could possibly influence phenology (l. 379).***

Reviewer #2: In this report, the authors use basic species distribution models and simple phenology models to extrapolate the current distribution and phenology of three culturally important shrubs species in a warmer future. While the study is generally clearly written and the methods are presented concisely, I feel that it lacks some novelty, as, to a large part, it seems to be quite similar to recently published study on huckleberry (AFM 2109, by the same authors. Referenced in this manuscript) just with different species three other different species.

***We appreciate the reviewer’s comment and recognize the similarities between the manuscripts. However, we note that including the detailed methods (as we did for the AFM 2019 manuscript) as well as the results for all four shrub species in one publication resulted in an unwieldly, extremely long manuscript that was challenging for readers to follow. Thus, we broke up the studies to allow for more discussion of the specific results for the various shrub species. This approach of breaking large studies into multiple manuscripts is not uncommon. We also note that novelty is not a requirement of publication in PLOS One.***

As such, the question remains if the authors exhaustively searched for all available data or, if even there is still more data on further species available, e.g. to cover all shrub species of the western US.

***There is an impressive amount of species location data available for Northwestern shrubs, and almost all relatively common species of plants and animals available on GBIF.org, and availability is expanding daily. However, modeling habitat suitability and phenology for all shrub species in the western US was beyond the scope of our research as we wished to focus on those species that were identified as being of significant cultural importance in the Northwest. We also want to underscore the time and computational expense involved in adding each additional species. Each species required a unique habitat suitability model to be developed using MaxEnt, and for each citizen scientist observation for each species, time was taken to view and verify that the species and phenology stage had been identified correctly before thermal sum models for species-specific phenology could be developed. So while the principal reason we constrained our analysis to three shrubs is a function of the identified cultural importance, the realities of the limitations on our time would have limited our analysis to far fewer than all shrub species across the western US even in the absence of our culturally-focused motivation for shrub selection. That said, we hope that our*** ***research will spur more efforts to monitor and model habitat and phenology changes of other shrub species.***

It would also be interesting ton include the results from the previous study into the current analysis and focus on the differences between the four species.

***This is also beyond the scope of our current work, and since the previous results for huckleberry are already published, we do not want to present the results again here. However, we’ve now added comparisons to our results for habitat suitability for the current species and huckleberry here (l. 312-314) and discuss differences in phenology between the three phenologically diverse shrub species in this manuscript in the Discussion (l. 334-338).***

Apart from that, my main critique is, that the authors seem to analyze and discuss the species distribution and phenology separately. I would like to see a deeper discussion of how a shifted phenology might interact with the bioclimatic parameters selected for the species distribution modelling in the long run, especially when making prediction for the end of the century.

***We now expand our discussion of how shifts in habitat suitability and phenology may interact in the Discussion (l. 400-405).***

Are the currently selected bioclimatic parameters still valid in a future climate with strongly shifted phenology? For example, are late winter freezing spells becoming a more of a problem when the phenology is shifted much earlier?

***This is a good point. We now expand our discussion of how shifts in habitat suitability and phenology may interact to increase frost risk of shrub species expanding into higher altitude and latitude ecosystems (l. 4002-405).***

Furthermore, the author claims that due to obvious limitations of the chosen very simple models (for which they provide a good rationale), this is just a first step to predict the responses into the future. With the authors being aware of the limitations, it would be important to discuss the most likely responses with more realistic models and their consequences for their predictions as well, also given that the direction in which the additional factors may influence the responses can be estimated.

***We have added several sentences to the discussion detailing how changes in other environmental controls over phenology would alter our results. L 380-384.***

All in all, it is a brief, solid study, but needs a major revision of the discussion, which in turn, could also lead to a much stronger conclusion.

***We thank the reviewer for their suggestions and agree that the revisions add substantially to the discussion.***

Some minor details:

The dataset is not available yet, doi missing.

***We will add the DOI to the data release as soon as the data is approved through the USGS data release review process, and prior to publication of the manuscript.***

There are a few wrongly formatted references in the introduction

***We have corrected the references in the Introduction (l. 58).***
