# Supplementary material for: Projected impacts of climate change on the range and phenology of three culturally-important shrub species
Source: PLoS One. 2020 May 8;15(5):e0232537. doi: 10.1371/journal.pone.0232537 (PMC7209123; doi:10.1371/journal.pone.0232537)
Supplement: Attachment — Submitted filename: response to minor revisions.docx [file pone.0232537.s009.docx]

4/10/2020

Dear Sergio Rossi,

We have addressed the minor revisions that you suggested, with specific changes in response to each point **in bold** below.

Thank you very much for your consideration of our manuscript.

Sincerely,

Janet Prevey on behalf of all authors

*Responses to editor comments:*

Discussion could benefit to be separated in sections of specific topics identified by subtitles.

**We have now separated the Discussion into topics identified by the subtitles ‘Species distribution’, ‘Phenology’, ‘Caveats’, and ‘Importance’.**

Consider to convert the last part of discussion in application. Or add a conclusion f the work.

**We’ve converted the last part of the Discussion into a subsection titled ‘Applications’.**

References are too many for a research paper. Generally, 30-40 should be enough. Reduce redundant or marginal references.

**We kindly request that we retain all the references for our manuscript. Since this research paper includes both climate envelope and phenology models, we have twice the number of necessary references to provide information for both disparate types of Methods, and many of these references were added per earlier reviewer comments. Additionally, 14 of the references are for the publicly available open-source data we used in the manuscript and are therefore necessary to include. We were not aware of any instructions to authors limiting the number of references for research papers in PLOS One.**

Readers should understand figures without reading the legend. As much as possible, improve the figures with this aim. Some examples: Fig 1: replace A, B, C with the name of the species, quantify (add values) to the range low-high, add the location of the study area at least in the legend; Fig 2: include the names of the scenarios (and the species)

**We have altered the Figures to replace the letters for panels with species names and the names of the scenarios, and added the location of the studies to Figure legends. In Fig. 1, the range values are denoted as ‘low’ and ‘high’ rather than 0 and 100 because, in Maxent, the computation of the cumulative output from the raw output means that large variations in cumulative value do not necessarily equate to large variations in the relative probability of presence, and thus it is advisable to list qualitative rather than numerical data, since a value of 100% does not necessarily mean that there is 100% chance of the species occurring at that location (Phillips and Dudik 2008). We do, however, quantify numerical differences in cumulative outputs for Fig 2.**

**Phillips SJ, Dudík M. Modeling of species distributions with Maxent: new extensions and a comprehensive evaluation. Ecography. 2008;31: 161–175.**
